# Cutaneous Melanocytic Tumor With CRTC1::TRIM11 Fusion: A Case Report

**DOI:** 10.7759/cureus.99193

**Published:** 2025-12-14

**Authors:** Ngoc Nguyen, Nathan Silvera, Jagadeesh Kumar

**Affiliations:** 1 Medical Education, Philadelphia College of Osteopathic Medicine, Philadelphia, USA; 2 Dermatology, Elkton Dermatology, Elkton, USA

**Keywords:** atypical epithelioid cells, cmtct, crtc1::trim11 fusion, cutaneous clear cell sarcoma, cutaneous melanocytic tumor, molecular analysis

## Abstract

Cutaneous melanocytic tumor with *CRTC1*::*TRIM11* fusion (CMTCT) is a rare and recently recognized melanocytic neoplasm with fewer than 50 reported cases. These tumors can be diagnostically challenging due to overlapping histologic features with malignant melanoma and clear cell sarcoma (CCS). This report adds to the limited literature by describing a 50-year-old male with an atypical inflamed cystic lesion on the shin. Biopsy revealed a dermal proliferation of atypical epithelioid cells within a desmoplastic stroma. Immunohistochemistry was positive for SRY-box transcription factor 10 (SOX-10) and microphthalmia-associated transcription factor (MITF), confirming melanocytic differentiation. Due to morphologic overlap with CCS, the specimen was referred for advanced molecular analysis, which confirmed a *CRTC1*::*TRIM11* fusion. The lesion was completely excised, and the patient underwent close dermatologic surveillance and follow-up. This case adds to the recognized clinical spectrum of CMTCT. We highlight the diagnostic complexity and role of molecular testing in distinguishing CMTCT from more common and aggressive neoplasms. Increased awareness among dermatologists and dermatopathologists can promote timely recognition and accurate diagnosis. The goal is for the expanding body of knowledge on CMTCT to continue informing diagnosis, long-term management, and providing insight into prognosis.

## Introduction

Cutaneous melanocytic tumor with *CRTC1*::*TRIM11* fusion (CMTCT) is a rare and emerging entity with fewer than 50 reported cases to date [1]. These tumors are diagnostically challenging due to their overlapping histologic and immunophenotypic features with other melanocytic lesions, particularly cutaneous clear cell sarcoma (CCS), which was previously referred to as melanoma of soft parts. This report describes an atypical presentation of CMTCT and adds to the limited body of literature on this rare entity. We highlight its diagnostic complexities, clinical implications, and the importance of molecular testing for accurate classification.

## Case presentation

**Figure 1:**
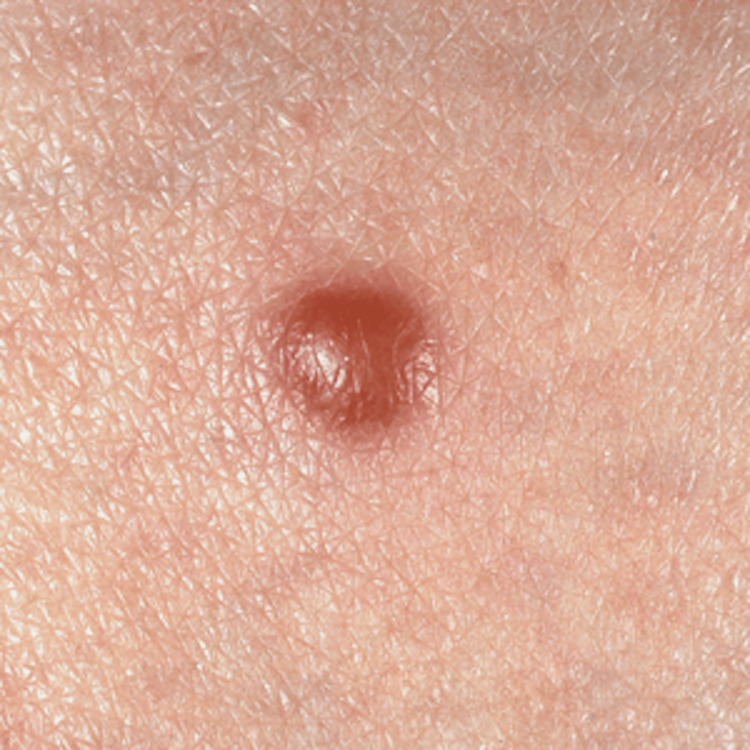
A 2.5-cm, round, soft, red to flesh-colored, well-circumscribed papule on the anterior shin of a 50-year-old man.

A 50-year-old male presented to the clinic with a soft cystic lesion on his left shin. The lesion was large and inflamed. It measured 2.5 cm in diameter and 1.5 cm above the skin surface (Figure 1). It was asymptomatic but cosmetically concerning. Given the cystic appearance, incision and drainage were attempted, but yielded no fluid. A 5-mm punch biopsy was then performed.

Histopathological evaluation demonstrated a dermal tumor composed of atypical epithelioid cells clustered within a desmoplastic stroma. Immunohistochemical staining was positive for SRY-box transcription factor 10 (SOX-10) and microphthalmia-associated transcription factor (MITF), supporting melanocytic differentiation. Due to diagnostic challenges and resemblance to CCS, the specimen was sent to the National Institutes of Health/National Cancer Institute for further molecular analysis, including methylation profiling and DNA and RNA sequencing. RNA sequencing confirmed a *CRTC1*::*TRIM11* fusion, establishing the diagnosis of CMTCT. The lesion was completely excised, and the patient was referred to the Johns Hopkins multidisciplinary surgical and medical oncology clinic. Given the lack of information on the long-term clinical course of CMTCT, the patient continued with close follow-up and dermatologic surveillance.

## Discussion

Cutaneous melanocytic tumors with *CRTC1*::*TRIM11* fusion often present as a slow-growing dermal or subcutaneous nodule, most commonly found on the extremities, followed by the trunk [2-3]. It affects a broad age range, with a median age of 43, and has no apparent gender predilection [1]. Grossly, CMTCT appears as a single well-circumscribed flesh-colored nodule but may exhibit inflammation or secondary changes that create a misleading clinical impression. Histologically, CMTCT is composed of atypical epithelioid, ovoid, and spindled cells clustered within a desmoplastic stroma. These cells are usually arranged in nests, bundles, or fascicles and contain prominent nucleoli with abundant pink cytoplasm [1].

CCS is the closest histologic mimic of CMTCT. CCS is a spindle cell neoplasm that generally arises in deep soft tissue but can occur intradermally [1]. Historically termed malignant melanoma of soft parts due to its morphologic and immunohistochemical similarities, CCS is now recognized as distinct from melanoma. It most often affects the extremities of adolescents and young adults and follows a slow but aggressive course, with a 20-year survival rate of approximately 10% and frequent recurrences and nodal metastases. CCS can be distinguished from CMTCT by its deeper anatomic location, multinucleated giant cells, and occasional clear cell morphology [1-2].

In a series of 40 CMTCT cases, half were initially misdiagnosed as CCS until genetic testing confirmed the diagnosis of CMTCT [1]. This underscores the importance of molecular characterization in distinguishing between the two entities. CMTCT is defined by a *CRTC1*::*TRIM11* in-frame translocation, while CCS harbors either *EWSR1*::*ATF1* or *EWSR1*::*CREB1* cytogenetic translocations [1]. Routine melanoma-focused next-generation sequencing (NGS) panels do not typically include *CRTC1* or *TRIM11*, so diagnosis often requires targeted RNA sequencing or fusion-based NGS assays [2]. Awareness of CMTCT can guide appropriate testing and facilitate prompt diagnosis.

Currently, CMTCT is treated with complete surgical excision. Definitive treatment guidelines have not been well established due to the novelty of the tumor [4]. Most cases follow an indolent course; however, the tumor has the potential to metastasize [1,4]. Reported cases include local recurrence, metastasis to regional lymph nodes, lung metastases, and aggressive clinical behavior in a subset of patients [5-6]. Due to the unpredictable nature and the scarcity of long-term data, regular and long-term dermatologic follow-up is necessary to elucidate the prognosis of this rare neoplasm.

## Conclusions

This case describes an atypical cystic presentation of CMTCT in a middle-aged male, adding to the growing body of literature and expanding its recognized clinical spectrum. Dermatologists and dermatopathologists should remain alert to CMTCT and its potential mimics. Maintaining a high index of suspicion when evaluating ambiguous cutaneous lesions, along with prompt consideration of molecular testing, is essential to ensure accurate diagnosis. Given the absence of standardized management guidelines and variable clinical presentation of CMTCT, treatment should be individualized. The long-term clinical course of CMTCT is unclear, and vigilant surveillance is critical for the timely detection of recurrence or metastasis.
